# Notes on Surgical Jaundice
*Communicated to the Bristol Medico-Chirurgical Society on 11th May, 1932.


**Published:** 1932

**Authors:** C. A. Moore

**Affiliations:** Surgeon, Bristol General Hospital


### NOTES ON SURGICAL JAUNDICE.*

BY

C. A. Moore, M.S. (Lond.), Ch.M., F.R.C.S.,
*Surgeon, Bristol General Hospital.*

---

I have no intention of attempting any exhaustive study of the subject of jaundice; I merely offer some remarks on the subject as seen solely from the surgical point of view. Thus I deliberately exclude from consideration all cases of jaundice due to any cause which may be roughly called medical, as well as any consideration of the pathological aspects, interesting though they are.

When a case of jaundice is seen by a surgeon, either in private consultation or on admission to a surgical ward, the cause is nearly always found to lie between three possibilities. These are: (*a*) obstruction of the common duct from within by a stone; (*b*) obstruction of the same duct, also from within but by some cause other than a stone, usually a primary growth of the ampulla; or (*c*) obstruction of the duct from without by the pressure of a pancreatic lesion, usually a carcinoma. A fourth form of obstruction affects the hepatic duct, most commonly by secondary growth in the portal fissure. In the differential diagnosis of these conditions careful attention to

* Communicated to the Bristol Medico-Chirurgical Society on 11th May, 1932.

points in the history will often suggest the correct diagnosis.

In common duct obstruction by a stone there will be the usual long history of flatulent dyspepsia, distinct variation in the depth of the jaundice, often from day to day, together with considerable rises of temperature, often accompanied by rigors. There may have been attacks of colic in the past when the cystic duct was being negotiated by the stone, but severe pain when the stone is in the common duct is not usual. It is well known, of course, that a stone in the common duct does not necessarily cause even jaundice.

When the common duct is obstructed from without by a pancreatic carcinoma the history differs in several respects. The jaundice comes on without pain or discomfort, sometimes after a week or two's diarrhœa; it steadily deepens without sign of remission; temperature is normal or subnormal and no rigors occur. In jaundice of this type, in consequence of its being deeper and more prolonged, itching of the skin is more constant and more troublesome than in calculous obstruction. No deduction can be drawn from the presence of wasting; jaundice itself, without relation to the cause, leads to more rapid wasting than almost any other condition. Wasting, then, is not in itself any reason for suspecting a malignant cause for the jaundice.

In these two classes of common duct obstruction there are thus strong points in the history which will often enable a diagnosis to be made with reasonable confidence, and when one turns to the physical signs the correct diagnosis may be still more clear.

In the case of gallstone obstruction it is well known that the gall-bladder is hardly ever anything but shrunken, and beyond the enlarged liver physical examination reveals nothing.

In pancreatic obstruction one will find, in a large proportion of cases, palpable evidence that the gall-bladder is considerably enlarged ; if it is, stone can be excluded with reasonable certainty. If it is not palpable in a case which otherwise suggests pancreatic obstruction, there may be two alternative explanations. One is that the gall-bladder is dilated, though not palpably so, because the liver is proportionately larger still, and conceals it ; the other that the case is one of hepatic duct obstruction, in which case the gall-bladder is, of course, not dilated.

In differentiating so sharply between these various types of obstruction I do not pretend that the diagnosis is always so simple ; in not a few cases there are points in the diagnosis that contradict one another in a most confusing way. If this is so, my experience is that when in serious doubt the cause is much more likely to be malignant than a stone.

I hope I have not devoted too much time to somewhat elementary points about the diagnosis, for it is to treatment that I want more particularly to refer.

Malignant obstruction of the hepatic duct permits of no treatment other than symptomatic. In the case of a stone in the common duct, wait for a remission of the jaundice, which will surely come, and remove the stone or stones, generally with the gall-bladder as well.

I want to deal in some detail with the treatment of cases where the jaundice is due to a carcinoma of the head of the pancreas. When these patients are seen, especially in hospital practice, they have often a deep jaundice of six weeks' standing; they are advanced in years, considerably wasted, and often, at first sight, almost hopeless operative propositions.

The treatment, of course, can only be palliative; radical treatment of carcinoma of the pancreas is usually out of the question. The operation consists in short-circuiting the obstructed bile-duct by anastomosing the dilated gall-bladder to some part of the gastro-intestinal tract—the duodenum if possible, which is often not the case; failing that, the stomach, colon or jejunum in that order of preference. The anastomosis with the stomach is technically easy, physiologically not unsound, and in practice gives excellent results.

This brings me to a point which I would especially emphasize: it is extremely unwise to attempt to complete the anastomosis in a deeply-jaundiced patient in a single-stage operation. It is well known to everybody that the presence of jaundice, if deep, adds markedly to the mortality of any operation; in a case of stone in the common duct, one waits confidently for an early recession of the jaundice. In a pancreatic obstruction no such remission can be produced by any natural process, but the surgeon can, by a comparatively small operation, rid the patient of his jaundice; ten days after a cholecystostomy the jaundice will have disappeared, and at the end of that time the small wound can be reopened and the fundus of the gall-bladder anastomosed to the pyloric end of the stomach. The disturbance is very small, and the general peritoneal cavity scarcely opened at all. It has been my experience that this operation is made much safer by so doing it in two stages.

It has been pointed out that the liver cells suffer severely from the effects of back-pressure when the common duct is obstructed, no less than do the renal cells in a comparable condition, *i.e.* in prostatic obstruction. No one would now do a single-stage operation for enlarged prostate at the end of six weeks' complete retention of urine.

In my opinion the case of common duct obstruction is strictly comparable. Although I have never, so far as I can remember, seen this point referred to in print, I make no claim to originality for it; in fact, I definitely disclaim it. I got the idea from Professor Burgess, of Manchester, in a paper he read at Bath some years ago. The idea struck me as essentially sound; since he gave me the idea I have never done a short-circuit in a deeply-jaundiced patient in one stage, and I shall never do it again.

One more point about these cases. They are, as I have said, in many cases and at first sight, almost hopeless operation risks, and some seem scarcely worth attempting. I believe that none should be refused the benefits of surgery without very careful consideration; if they are done in two stages it is surprising what apparently hopeless cases can be saved. If they can be rid of their jaundice the operation will be well worth while, especially if they have itching with it, as most have. It has been said that the mortality from suicide in the worst itching cases is higher than the operative mortality; this may be an exaggerated statement, but it is not without a shadow of truth. At any rate, a patient who can be rid of his jaundice can expect many months of comparative comfort and a painless end, quite a different story from the final weeks of one with deep and increasing jaundice.

Before I close I should like to exemplify my remarks by a brief reference to three cases, about each of which there are points of interest.

The first was a lady, aged 61, but looking more. She had had deep jaundice for two months, and I diagnosed carcinoma of the pancreas and advised short-circuiting. This was refused until her husband, who was a doctor, had taken her on a medical tour of London, seeing in one day a well-known physician, an equally well-known surgeon, and visiting the Radium Institute. None of these authorities could offer any better suggestion than mine, and she returned to me.

I opened her abdomen, and found a thin, distended gall-bladder and a hard mass in the head of the pancreas. I drained her gall-bladder and she lost her jaundice, although she was rather slow in picking up. Three weeks later I completed the short-circuit, and she made a satisfactory recovery.

The after-history of this case is not without interest. After a short interval to convalesce the patient went for a seaside holiday, and the day before she went she rather staggered me by asking if I had any objection to her indulging in sea-bathing, which I sanctioned. When she returned she told me with great gusto that she had bathed every day except one, when it was too rough and stormy. She had, however, evened things up by bathing the following day before breakfast as well as at her usual time! She went on quite comfortably, looking after her house and doing her shopping on foot, for some months. I saw her for the last time just over a year from the operation, obviously failing fast, with a large abdominal tumour and some ascites, but no jaundice. She died a fortnight later.

The second case is that of a woman of 30—the youngest case of carcinoma of the pancreas I have ever seen. She had the usual signs and symptoms and I confirmed the diagnosis by an operation, doing a two-stage anastomosis. She lost her jaundice and made a rapid recovery, so complete that, in view of her age, I naturally doubted the accuracy of my diagnosis. She returned to her work as a domestic servant, and I lost sight of her until many months later I saw her again, now very much wasted and with a large mass in her epigastrium. She died shortly after.

The third case was that of a woman of 46, on whom I operated in December, 1926. She had the usual history and physical signs and was deeply jaundiced. My note, taken at the time, states that she had a mass in her pancreas which was "evidently malignant." I did an anastomosis between her gall-bladder and stomach; she lost her jaundice and made a good recovery.

I heard of her several times in the following years, being told by her doctor that she was quite well, free from jaundice, but with an epigastric swelling of considerable size. I did not see her myself until January of the present year, when she appeared with no jaundice or epigastric swelling, looking quite well, but complaining of a swelling in her neck. This proved to be a single hard gland in the left posterior triangle, not indeed supraclavicular but rather in the upper part of the triangle. I excised this gland, expecting to find growth in it, but this was not the case. It was reported as being full of giant cells and presumably tuberculous.

I saw her again a few weeks later, this time with a swelling in the infraspinous region of her left scapula; the swelling had all the signs of a metastasis, and I thought it must be one. Again I excised it, and found a necrotic-looking mass, involving the muscle but not the bone. The pathological report said it was probably gummatous, an opinion that was confirmed by a strongly positive Wassermann reaction.

It is possible, or probable, that the original pancreatic lesion was of the same nature. If I find myself compelled to apologise for taking five and a quarter years to make the diagnosis, I feel equally entitled to congratulate myself on having saved her life by operating, for she would certainly have died of the biliary obstruction, and her death would have been certified as due to carcinoma of the pancreas.

To sum up my ideas on this question, I hold :—

1. That no case of obstructive jaundice should be refused operation without due consideration of the pros and cons.

2. That the operation in deeply-jaundiced patients should be a two-stage one.

3. That the relief obtained in successful cases is well worth the risk. Death, even at the surgeon's hands, is preferable to the only alternative—a few weeks of misery with death coming as a welcome deliverance.

4. That any case of jaundice which has lasted without remission for two weeks is potentially surgical. It is only by the realisation of this that we shall avoid unjustifiable delay and be able to give the maximum of relief to sufferers from this condition.

